# A case report: acute pancreatitis associated with tacrolimus in kidney transplantation

**DOI:** 10.1186/s12882-019-1395-x

**Published:** 2019-06-07

**Authors:** Junnan Xu, Liang Xu, Xing Wei, Xiang Li, Ming Cai

**Affiliations:** 1 Organ Transplant Institute of People’s Liberation Army, the 309th Hospital of People’s Liberation Army, Beijing, China; 2 Beijing Key Laboratory of Immunology Regulatory and Organ Transplantation, Beijing, China; 3 Medical School of Chinese PLA, the Chinese PLA General Hospital, Beijing, China

**Keywords:** Acute pancreatitis, Tacrolimus, FK506, Kidney transplantation

## Abstract

**Background:**

Tacrolimus has been widely used for immunosuppressive therapy in solid organ transplantation (SOT) and allo-geneic stem cell transplantation (allo-SCT) over the past 2 decades. Pancreatitis caused by tacrolimus was rarely reported in kidney transplantation previously.

**Case presentation:**

Here we presented a case of a 45-year-old male who underwent kidney transplantation and received immunosuppressive therapy of tacrolimus, on day + 67 after transplantation he developed acute pancreatitis with extremely high blood concentration of tacrolimus. We excluded other possible causes and speculated tacrolimus was the probable inducer of pancreatitis. After tacrolimus was discontinued and alternated with cyclosporine, he gradually recovered and was discharged home with no relapse.

**Conclusion:**

Tacrolimus can be a probable cause of pancreatitis after kidney transplantation. We recommended clinicians to be aware of the possibility of tacrolimus-induced pancreatitis during tacrolimus treatment.

## Background

Drug-induced pancreatitis is an adverse effect associated with varies of drugs, more than one hundred drugs have been reported to be the probable causes of pancreatitis [1, 2]. Pancreatitis, reported in SOT and allo-SCT cases, was considered to be related with immunosuppressive agents. However, only azathioprine has been confirmed to be the cause of pancreatitis with solid evidence so far [3].

Tacrolimus is a commonly used immunosuprressant with variety of adverse effects, including neurotoxicity, hepatotoxicity, nephrotoxicity, infection and so on. However, very few cases that associated pancreatitis with tacrolimus were reported, especially in kidney transplantation [4–7]. Here, we presented an additional case of pancreatitis in a male kidney transplant recipient who received tacrolimus treatment after transplantation.

## Case presentation

The patient was a 45-year-old male who was admitted to our institution due to uremia. He had no diabetes and biliary tract disease history, and his BMI (body mass index) was 22.99 kg/m^2^ (183cm, 77 kg). He underwent kidney transplantation in our institution on 9th August, 2017. The donor was from donation after cardiac death (DCD). Before surgery, he received antilymphocyte therapy of basiliximab (20 mg i.v.). The surgery was successful and the initial immunosuppressive regimen consisted of tacrolimus (6 mg/day, 0.078 mg/kg/day), mycophenolate mofetil (1500 mg/day) and corticosteroids (initial dose 35 mg/day). The patient recovered well after surgery and was discharged on day 26+ with blood creatine level 156.6umol/L and trough concentration of tacrolimus 10.6 ng/ml then. After discharged, He reexamined in our institution once a week. From day 26+ to day 60+, the reexamine results showed his blood creatine level continued to decline to 101.7umol/L (day 60+), the dosage of corticosteroids was gradually tapered from 35 mg/day to 5 mg/day, and the dosage of tacrolimus was maintained at 6 mg/d with trough concentration ranged from 9.5–11.2 ng/ml. In addition, the recipient neither had a history of high fat diet nor presented hyperlipidemia from day 1+ to 67+ posttransplant, the laboratory analysis results showed the serum triglyceride (TG) level was in the range of 0.71–1.43 mmol/L while the cholesterol (CHOL) level was 3.3–4.5 mmol/L during the period.

**Fig. 1:**
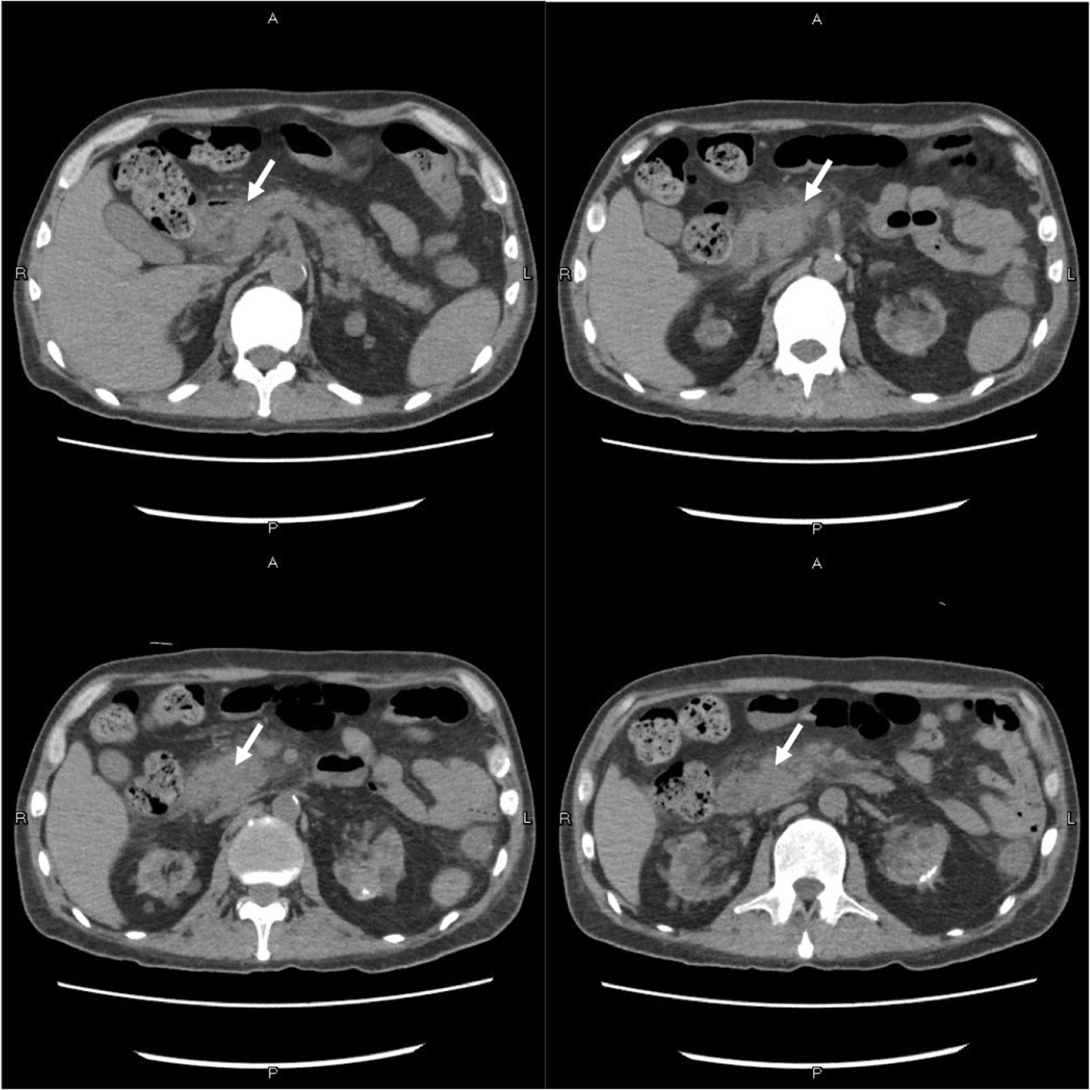
Abdominal computed tomography scan showed swelling pancreatic head and peripancreatic inflammation (white arrow)

On day 67+, he presented with acute abdominal pain in middle and left area of abdomen accompanied with nausea and vomiting. Physical examination showed diffuse abdominal tenderness with diminish bowel sound. Laboratory analysis showed WBC 9.16 × 10^9^/L, neutrophils 7.98 × 10^9^/L, hemoglobin 73 g/L, platelets 78 × 10^9^/L, blood creatinine 147.4umol/L, blood urea nitrogen (BUN) 17.79 mmol/L, calcium 2.52 mmol/L, potassium 4.72 mmol/L, sodium 138 mmol/L, serum amylase 679.3 IU/L (normal 15–115), lipase 755 U/L (normal 6–51), trough concentration tacrolimus> 30 ng/ml (dosage was 6 mg/day) accompanied with elevated fasting blood glucose (29.49 mmol/L) and diabetic ketoacidosis (DKA) tendercy (blood ketone body test +). Abdominal computed tomography scan showed enlarged pancreatic head with peripancreatic inflammation and inflammatory exudate without dilatation of biliary tract (Fig. 1). Pancreatitis diagnosis was made on the basis of the information all above.

Tacrolimus treatment was discontinued, somatostatin (3 mg, q12h, i.v.) was administrated to treat pancreatitis, other treatments included fluid replacement, insulin (i.v.) and imipenem (1 g, q12h, i.v.). Three days after somatostatin using (day 70+), patient’s condition improved significantly, abdomen symptoms relieved, blood amylase level were normalized (72 IU/L) and lipase level deceased to 69 U/L, blood glucose level maintained around 10–15 mmol/L. Cyclosporine (4 mg/kg/day) was administrated as an alternative for tacrolimus on day 75+, and the patient was discharged on day 80 + .

There was no recurrence of pancreatitis in 7 months follow up.

## Discussion and conclusions

Pancreatitis after SOT or allo-SCT have been associated with immunosuppressive treatment previously. However, among the immunosuppressive drugs commonly used, only azathioprine was proved to be the definite cause of pancreatitis [1]. Though tacrolimus has been widely used in transplantation over the past two decades, pancreatitis induced by tacrolimus was rarely reported. In kidney transplantation, only one case was reported by Ogunsiende et al. suggested tacrolimus was the probable cause of acute pancreatitis [4]. Other cases were from heart, liver and allogeneic umbilical cord blood transplantation [5–7].

In our case, at the time onset of pancreatitis, the patient was receiving varies of drugs including tacrolimus, mycophenolate mofetil, corticosteroids, calcitriol, rabeprazole, nifedipine. Besides tacrolimus, only corticosteroids were reported to be the probable cause of pancreatitis. However, the dosage of corticosteroids was kept at low level (5 mg/d) when acute pancreatitis was presented. Moreover, pancreatitis was relieved and did not relapse with corticosteroids withheld. Therefore, corticosteroids in this case can be reasonably excluded as the cause of pancreatitis.

Other possible causes can also be excluded in this case. Extrahepatic biliary obstruction, hypercalcemia and hyperlipidemia were not observed in CT scan and laboratory examination. No evidence of CMV (cytomegalovirus), zoster, adenovirus or other virus infections was presented. Drugs with definitive association with pancreatitis such as sulfonamides, pentamidine, furosemide was not administrated at the time pancreatitis was presented.

Mallory and Kern established a criterion to identify the association of pancreatitis with any drug. The criteria included:1) appearance of pancreatitis during treatment with the drug; 2) disappearance upon withdrawal of the drug; 3) exclusion of other causes; 4) relapse upon rechallenge [8]. According to Mallory and Kern’s criteria, our case meets the first 3 criteria. Since tacrolimus was not readministrated, rechallenge was not available in this patient. Moreover, extremely high trough concentration of tacrolimus coincided with the onset of pancreatitis. Therefore, we consider this case as a probable association between pancreatitis and tacrolimus.

To our knowledge, this is the second report of tacrolimus-induced pancreatitis in kidney transplantation. Although pancreatitis has not been established as an adverse effect of tacrolimus, we would like to alert clinician to the possibility of tacrolimus-induced pancreatitis during tacrolimus treatment.

## Data Availability

All data supporting our findings are contained within the manuscript.

## Abbreviations

allo-SCT: Allo-geneic stem cell transplantation
BMI: Body mass index
CMV: Cytomegalovirus
DCD: Donation after cardiac death
DKA: Diabetic ketoacidosis
DKA: Diabetic ketoacidosis
SOT: Solid organ transplantation
